# Prophylactic Tracheostomy for Inhalational Burns

**DOI:** 10.29252/wjps.9.1.10

**Published:** 2020-01

**Authors:** Abhinav Aggarwal, Ravi Kumar Chittoria, Vinayak Chavan, Chirra Likhitha Reddy, Saurabh Gupta, Padmalakshmi Bharathi Mohan, Imran Pathan, K Shijina

**Affiliations:** Department of Plastic Surgery, Jawaharlal Institute of Postgraduate Medical Education and Research (JIPMER), Pondicherry, 605006, India

**Keywords:** Prophylactic, Tracheostomy, Inhalation, Burns

## Abstract

**BACKGROUND:**

Various studies have reported different conclusions over the safety and benefits of early tracheostomy in burns. Our study aimed to assess the role of prophylactic tracheostomy in treatment and improvement of outcomes in inhalational burns in India.

**METHODS:**

In a retrospective descriptive analysis of burns admitted over 1 year in Jawaharlal Institute of Postgraduate Medical Education and Research (JIPMER) Tertiary Burns Center in India, patients with thermal burns of TBSA less than 60% and those with indirect evidence of airway burns were enrolled and divided into two groups who underwent prophylactic tracheostomy vs. patients for whom prophylactic tracheostomy was not done. Mortality was the final point and primary variable measurement.

**RESULTS:**

Totally, 10 patients with inhalational burns were admitted. Out of the 4 patients for whom prophylactic tracheostomy was undertaken, three patients survived, while one died. Out of the 6 patients for which prophylactic tracheostomy were not performed, 4 patients died; while 2 survived.

The average percentage of burns TBSA in the prophylactic tracheostomy group was 34%. Average age of patients in the prophylactic tracheostomy group was 31.3 years. The average percentage burns TBSA in the group, where prophylactic tracheostomy was not carried out was 42%. Average age of patients in the prophylactic tracheostomy group was 36.2 years.

**CONCLUSION:**

Our study is a pilot study to investigate the possibility and a way to improve outcomes in patients with inhalational injuries. Larger trials may be needed to facilitate or disprove the same.

## INTRODUCTION

In developing countries, burn injuries impose a great burden^1^^-^^3^ and is a common problem with incidence of 6-7 million per year in India.^4^ There are 140,000 mortalities and 700,000 patients required admission every year.^5^ Despite the widespread problem, progress of the treatment of major burns has failed to keep up with the advances of medical science in a country like India. There is a paucity of burn centers and specialized burn care. Airway management is one of the major management problems in thermal burns with laryngeal edema, airway injury, acute respiratory distress syndrome (ARDS), and pneumonia as the contributors in chief. The treatment of inhalation injury is mainly supportive and the mortality is high.^6^

Inhalational injury features as supraglottic thermal injury, chemical irrigation of the respiratory tract and systemic toxicity due to soot and carbon monoxide leading to an inflammatory response that leads to pneumonia, ARDS and death. The classical management of inhalation injury includes diagnosis by clinical markers like head and neck burns, nasal singing, presence of dyspnea, and hoarseness of voice with anatomical evidence of airway injury confirmed by fibro-optic bronchoscopy (FOB).^7^

Prophylactic intubation has been indicated in all cases with airway burns due to progressive airway obstruction caused especially by edema, when large volume resuscitation is indicated as happens in major burn injuries. Studies have suggested prophylactic intubation that would lead to a decrease in pulmonary related mortality of patients.^8^ Classical dictum of early tracheostomy went into disrepute and led to fewer tracheostomies replaced by early intubation and ventilation for the initial hospital stay.^9^

Tracheostomy has a number of practical benefits as compared to endotracheal intubation. It is far less irritating for the patient, permits maintenance of oral hygiene and early ambulation. Moreover, they are much more secure than endotracheal tubes and can be replaced easily by the nurse, if inadvertently dislodged.^10^ Tracheostomy aids early weaning off the ventilator and decreases the work of breathing. This study was undertaken to determine the role of prophylactic tracheostomy in treatment and improvement of outcomes in inhalational burns in India.

## MATERIALS AND METHODS

A retrospective descriptive analysis of burns admitted over 1 year was carried out in Jawaharlal Institute of Postgraduate Medical Education and Research (JIPMER) Tertiary Burns Center in Pondicherry, India. The patients were fitted into the inclusion and exclusion criteria. The inclusion criteria were (i) Thermal burns of TBSA less than 60% and (ii) Indirect evidence of airway burns must be present (Deep burns to chest, neck, face, abdomen, nasal hair singing, dyspnea, and change in voice). Burns more than 60% TBSA, superficial burn injuries, electric burn injuries, chemical burn injuries were excluded from the study.

All patients with all comorbidities contributing to the respiratory illness like cardiac disease, hypertension and bronchial asthma were excluded too. Patients fitting into the criteria were divided into two groups of patients who underwent prophylactic tracheostomy vs. patients in whom prophylactic tracheostomy was not done. Mortality was the end point and primary variable measurement. Rest of confounding factors like age, sex, percentage of burns was evaluated separately. Descriptive analysis was done.

## RESULTS

Totally, 10 patients with inhalational burns were admitted matching the inclusion criteria. Out of the cases, all patients were offered the option of prophylactic tracheostomy; but only 4 of them were consent to it. Out of the 4 patients for whom prophylactic tracheostomy was undertaken, three patients survived while one died. Out of the 6 patients for which prophylactic tracheostomy were not performed, 4 patients died; while 2 survived. 

In the group with prophylactic tracheostomy, 3 were males and one was female. In the group where prophylactic tracheostomy was not done, 4 were males and 2 were females. The average percentage burns TBSA in the prophylactic tracheostomy group was 34% (25% to 45% TBSA). Average age of patients in the prophylactic tracheostomy group was 31.3 years (15 to 54 years). The average percentage burns TBSA in the group, where prophylactic tracheostomy was not carried out was 42% (30% to 54% TBSA). Average age of patients in the prophylactic tracheostomy group was 36.2 years (21 to 56 years). No complications of tracheostomy were noted in our study.

## DISCUSSION

Despite the remarkable advances in management of burn patients, burn injuries continue to claim a high toll, particularly in predisposed patients and severe burns.^11^^,^^12^ Tracheostomy in burns is a controversial topic with no clear consensus.^13^^-^^15^ There is concern regarding the associated complications. Eckhauser *et al.*^9^ have reported an increased incidence of mortality and morbidity after tracheostomy, while Aggarwal *et al.*^10^ have demonstrated a better survival in tracheostomized burn patients following burn injuries. Tracheostomy benefits are ample in inhalation burn injury patients. These patients produce thick secretions in response to respiratory mucosal inflammation and tracheotomy felicitates better secretion clearance.

Complications of tracheostomy include hemorrhage, dysphagia, tracheal stenosis.^9^^,^^10^ None of these complications were seen in our study. Additionally dysphagia and dysphonia were documented complications of endotracheal intubation as well and the timing of tracheostomy is very critical.^9^^,^^10^ There have been studies that showed a survival benefit with early airway management.^9^^,^^10^ This is especially relevant for a country like India, where diagnosis of inhalation injury is generally missed due to lack of facility for bronchoscopy and specialized burn centers.

In our study, a lot of confounding variables like contributing causes to patient outcome have not been analyzed. Moreover due to a small sample size, significance cannot be commented upon. The diagnosis of inhalational injury was based only on clinical scenario, also lead to the addition of many patients in whom inhalational burns might not have been present. Nonetheless, the study showed that the group with prophylactic tracheostomy fared better in patients in whom early intervention was not done. This finding can certainly be kept in mind while managing similar cases in a resource limited scenario in developing countries. 

As literature states, the issue is a controversial one, with authors advocating tracheostomy and as the patient can be mobilized early, better oral hygiene and early rehabilitation can be noted; while others discouraging it due to increased rate of complications as compared to endotracheal intubation. Nonetheless, the study showed that the group with prophylactic tracheostomy fared better in patients in whom early intervention was not done. Our study was a pilot study to investigate the possibility and a way to improve outcomes in patients with inhalational injuries. Larger trials may be needed to facilitate or disprove the same.

## CONFLICT OF INTEREST

The authors declare no conflict of interest.
